# Comparative evaluation of ARV therapy efficiency among patients at the Kiev regional AIDS center

**DOI:** 10.1186/1758-2652-13-S4-P153

**Published:** 2010-11-08

**Authors:** YO Yurchenko, T Stepchenkova, KI Karnets, MO Martinenko

**Affiliations:** 1 Kiev regional AIDS center, Kiev, Ukraine; 2 Kiev regional AIDS center, Clinical laboratory, Kiev, Ukraine; 3 Kiev regional AIDS center, ambulance, Kiev, Ukraine

## Background

Ukraine takes one of the leading places in the growth of the epidemic of HIV infection/AIDS in Europe. Nowadays, more than 1.4% adult population is HIV infected. All people who need, receive ARV therapy. Today almost 12000 patients use it.

## Purpose of the study

Specialists of the Kiev regional AIDS center made a comparative evaluation of ARV therapy efficiency among patients at the center during the first half of the 2008 and the same period of 2009. They analyzed a number of virological and immunological faults in the treatment of the patients, who use ARV therapy during 1 year.

## Results

Number of tested patients:

- 924(2008)

- 952(2009)

Among them, number of patients who received ARV therapy during 1 year:

- 170(2008)

- 169(2009)

Number of faults (both virological and immunological) abs\% from all persons who received ARV therapy:

- 58\32%(2008)

- 53\31% (2009)

Structure and % of the faults to the whole number of tested patients:

1. Only virological faults:

- 3\1%(2008)

- 2\1%(2009)

2. Only immunological faults:

- 41\24%(2008)

- 31\18%(2009)

3. Both virological and immunological faults:

- 14\7%(2008)

- 20\12%(2009)

All patients, who had both virological and immunological faults of the therapy, were suggested to change the scheme of the therapy. The effectiveness of these changes was analyzed after 6 month by the same criteria:

• Detected viral load level

• CD4 cells level lower than 200 cells/ml.

Effective change of the scheme of the ARV therapy:

- 10\71% (2008)

- 12\60% (2009)

Faults of the changed scheme of the ARV therapy : - 3\21%(2008)

- 7\35%(2009)

## Conclusions

We can make a conclusion that in 2009 there were more both virusological and immunological faults as a result of the ARV therapy. It can be an evidence of the increasing of the number of the resistant viruses which are circulated in the population of HIV infected persons in Ukraine. This conclusion can be confirmed by the low effectiveness of the changing of the schemes ARV therapy. It can be a probable evidence of the formation of the multi-resistance variants of the viruses.

So this study shows the necessity of introduction of the researches of virus's resistance in the practice of laboratory researches for HIV infected people, who receive ARV therapy in Ukraine.

